# Heart Failure: A Deficiency of Energy—A Path Yet to Discover and Walk

**DOI:** 10.3390/biomedicines12112589

**Published:** 2024-11-12

**Authors:** Ioannis Paraskevaidis, Christos Kourek, Dimitrios Farmakis, Elias Tsougos

**Affiliations:** 1 6th Department of Cardiology, Hygeia Hospital, 151 23 Athens, Greece; iparas@otenet.gr (I.P.); tsougos@yahoo.com (E.T.); 2 Department of Cardiology, 417 Army Share Fund Hospital of Athens (NIMTS), 115 21 Athens, Greece; chris.kourek.92@gmail.com; 3 Heart Failure Unit, Department of Cardiology, Attikon University Hospital, Medical School, National and Kapodistiran University of Athens, 124 62 Athens, Greece

**Keywords:** heart failure, cardiac energy, starving heart, cardiac metabolism, cardiac biochemical substrate, mitochondria function

## Abstract

Heart failure is a complex syndrome and our understanding and therapeutic approach relies mostly on its phenotypic presentation. Notably, the heart is characterized as the most energy-consuming organ, being both a producer and consumer, in order to satisfy multiple cardiac functions: ion exchange, electromechanical coordination, excitation–contraction coupling, etc. By obtaining further knowledge of the cardiac energy field, we can probably better characterize the basic pathophysiological events occurring in heart disease patients and understand the metabolic substance changes, the relationship between the alteration of energy production/consumption, and hence energetic deficiency not only in the heart as a whole but in every single cardiac territory, which will hopefully provide us with the opportunity to uncover the beginning of the heart failure process. In this respect, using (a) newer imaging techniques, (b) biomedicine, (c) nanotechnology, and (d) artificial intelligence, we can gain a deeper understanding of this complex syndrome. This, in turn, can lead to earlier and more effective therapeutic approaches, ultimately improving human health. To date, the scientific community has not given sufficient attention to the energetic starvation model. In our view, this review aims to encourage scientists and the medical community to conduct studies for a better understanding and treatment of this syndrome.

## 1. Introduction

**Figure 1:**
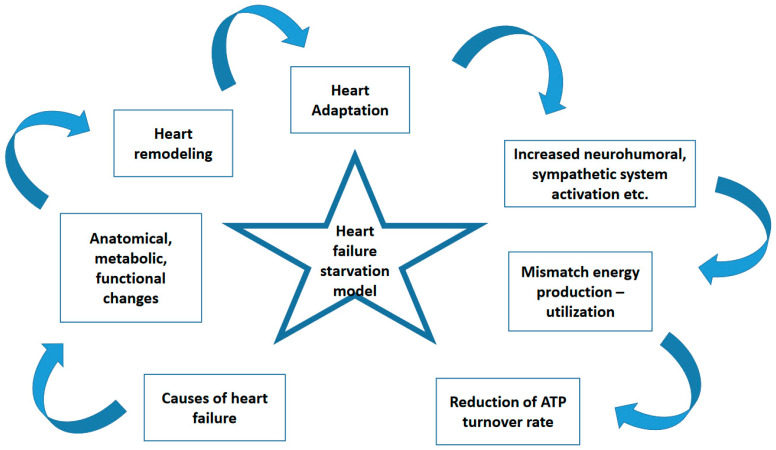
Heart failure starvation model. After an index event, anatomical, metabolic, and hence functional changes are observed, followed by cardiac anatomical remodeling, neuro-humoral adaptation, and a reduction in cardiac energetic status.

Heart failure is a syndrome that describes the inability of the heart, due to structural and/or functional abnormality, to satisfy the metabolic needs of the periphery under normal filling pressures. Despite this definition, which incorporates both volume and pressure variables [1], the guidelines, based on ejection fraction, suggest a phenotypic subdivision for diagnostic categorization and therapeutic purposes [2]. However, this commonly and widely used index, the left ventricular ejection fraction, has several drawbacks and is dependent on various parameters [1,3]; therefore, it is time to reconsider it and try to better understand the source and consequences of cardiac pathophysiologic alterations in patients with heart failure, as shown in Figure 1. Indeed, it seems that insisting on treating heart failure based mainly on phenotypic subdivision, despite the steps forward that have been taken, is not the most appropriate mode of action, since the morbidity and mortality rate remains high. The volumetric and pressure variables involved—affected regardless of the cause of heart failure—are the promoters of sympathetic activation, renin–aldosterone axis activation, free radical production, inflammation, and overactivation. The heart does not respond as a whole to these variables, but any single cardiac segment reacts accordingly with a time gap. In fact, there are different wall layer mechanics, along with an interplay between the base and apex, acting in such a way as to successfully coordinate the sequence of diastole and systole [4]. For example, during isovolumic contraction, sub-endocardial right-handed helical fibers start earlier than sub-epicardial left-handed helical fibers, later coinciding with left ventricular ejection [5], suggesting a non-simultaneous involvement of each segmental territory of the heart. To ensure that this coordination is continuous, each segment of the heart is obliged to produce active mechanical force in order to fulfill the main goal. This force incorporates several forms of energy including kinetic energy, wall–volume motion; dynamic energy, intra-ventricular pressure variance; thermal energy, etc., and it seems that the energy interplay during the cardiac cycle constitutes the basic pathophysiologic status of the heart. Energy is needed for multiple cardiac functions: ion exchange, electromechanical coordination; excitation–contraction coupling, etc. Indeed, contraction, relaxation, torsion, twisting and untwisting, thickening, and thinning are movements of the heart that require energy and that are, over time, altered in patients with heart failure. Accordingly, although the total energy remains constant, the percentage of energy contribution of each subset is altered. The mechanical energy, both dynamic and kinetic, is decreased and hence is unable to adequately achieve the cardiac functions [6]. In this respect, there is a continuous change in the micro and macro architecture of the heart, trying to maintain the correct cardiac cycle in order to fulfill the metabolic needs of the periphery [7]. Interestingly, as mentioned before, these changes are not homogenously, uniformly, or simultaneously distributed to cardiac segments, raising questions as to whether the cardiac muscle is operating as an anatomical syncytium. Accordingly, it has been shown that the mechanical energy at the base of the heart is the key aspect of cardiac function at rest, whereas during exercise, the base at the phase of contraction and the apex during relaxation play the main roles [4]. This might suggest that in patients with heart failure, their limited exercise capacity might be due to the reduced energetic status of the base during the phase of contraction and of the apex during the phase of relaxation, both being unable to satisfy the total cardiac energy requirements. Therefore, we have to look not only at the overall cardiac functional activity but also at segmental energy contributors/users. Indeed, it seems that a regional segment energetic evaluation can teach us a lot [8], particularly about total cardiac function regardless of total energetic status, which at the end of the day may be normal but will worsen with time. Thus, abnormal regional cardiac energetic and metabolic resources might be an earlier sign of the ongoing process of heart failure. Moreover, both suction and expulsion forces created by counter-directional cardiac motion create a vortex pattern that is heterogeneously distributed in the heart [5]. Thus, we have to find the way to explore the needs of each cardiac segment and its capacity to properly use the produced energy. Production and consumption depend on cardiac metabolic resources and on the functional status of the cardiac silhouette including cardiomyocytes and non-cardiomyocytes cells. In this respect, it seems it is appropriate to search for the metabolic and energetic behavior of each segment and not for the global indices of heart function: ejection fraction, global longitudinal strain, total energy, etc.

### Phases of the Cardiac Cycle

For conventional reasons, the cardiac cycle is divided into two major phases; systole and diastole. Each period of these two phases is energy-dependent (except the diastolic relaxation period). Notably, the contraction–ejection and the early phase of isovolumic relaxation, the suction, are the periods with the highest energy demand and expenditure. These periods represent the connection between the heart and systemic–pulmonary circulation. In fact, to facilitate the blood volume flow to either direction, there is a need to create a drive pressure or pressure gradient. In the case of ejection, the contraction of cardiac muscle generates a pressure gradient between the left ventricle and the systemic circulatory resistance a gradient of over 60 mm Hg. In the case of cardiac filling, there is a gradient between pulmonary circulation and the left atrium of no more than 4–6 mmHg, and under these circumstances the blood flow is limited. Thus, in order to create a functional pressure gradient and hence a blood volume flow, at the suction period: early diastole, a negative intraventricular pressure is created. The normal systemic–pulmonary circulation is maintained on the expense of a highly energetic expenditure procedure. Under normal electromechanical cardiac function, the produced energy satisfies the energetic needs of the heart. However, when cardiac function is impaired, there is a need for additional actions and hence an additional energetic cost in any single step during the heart failure process: neurohumoral and sympathetic overactivation, inflammation, production of free radicals, mitophagy autophagy, etc.

As mentioned before, each ventricular segment during these two periods: ejection and suction, is in close continuity and working as a single unit, as a functional syncytium, which works at different time points. Moreover, any single cardiac segment during the same period acts in a non-homogenous way. This suggests a different energetic status in every cardiac segment at different time periods: ejection–suction, as well as during the same cardiac period [4]. Importantly, the segmental functional heterogeneity is changed physiologically in both normal and diseased individuals. Indeed, in patients with a preserved ejection fraction, the time of active relaxation corresponds to the time of ventricular shortening in normal individuals. Both actions have very energetic demanding properties [9]. The energetic force that mobilizes Ca^2+^ concentration, the exchange of being bonded and unbonded to the myofilament protein troponin, creates the contraction force and relaxation. The contraction force is made up by isometric force and rapid shortening with concomitant intra-cavity pressure creation and cardiac blood volume ejection, respectively. Indeed, during the heart filling with blood period, the myofilaments are stretched as a consequence of increased Ca^2+^ sensitivity and respond with stronger contraction, strongly dependent upon the energetic reserve of the syncytium. Following contraction, the Ca^2+^ must be removed from the myofilament and this is achieved in several ways: Na/Ca^2+^ exchange, sarcolemma Ca^2+^ ATPase pump, etc., all dependent on the energetic status. Importantly, during this period, Ca^2+^ may enter the mitochondrion in a rapid–high or slow–low way. If the rapid–high flow is the case, the overwhelming of mitochondria with catastrophic consequences occurs leading to depressed function and hence reduced energy production. In the case of slow–low flux, there is an increase in mitochondrial NADH concentration and hence ATP production to the extent necessary to fulfill the cardiac work and metabolic needs. Thus, the Ca^2+^ flux balance, its interplay with the sarcoplasmic reticulum and mitochondria, and the expected sympathetic activation needed in order to keep the whole procedure under control are small examples of the energetic needs for each myocardial cell to maintain inotropy and lusitropy, thus satisfying the main mission of the heart [10]. Interestingly, the sarcomere contraction is governed linearly by the rate of ATP consumption regardless of the Ca^2+^ cycle, and along with excitation–contraction coupling (ion exchange), indicates additional sources of energy expenditure [11]. At the same time, the homeostatic mechanisms expend energy to protect myofilaments and cytosol from free Ca^2+^ and ATP excess, as well as from the aggregation of other harmful products. 

## 2. Cardiac Fuel—Cardiac Energy Metabolism

**Figure 2:**
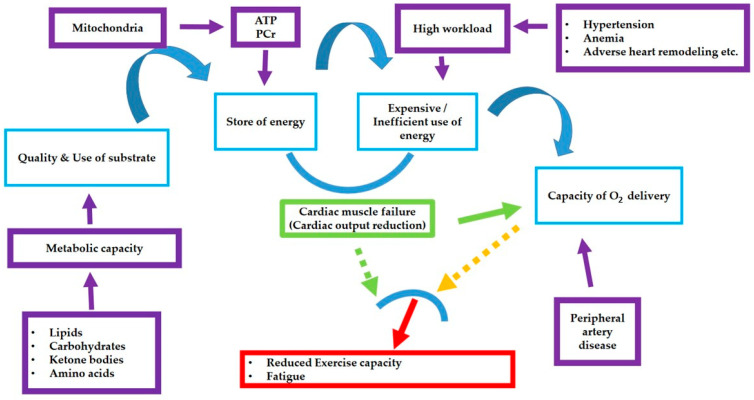
Insufficient use or production of energy due to decreased substrate metabolism, peripheral arterial diseases, mitochondrial defects, or high workload leads to reduced exercise capacity and fatigue.

The cardiac energy network is primarily based on two molecules: ATP and creatine phosphate (Pcr), governed by the equation [ATP]/[ADP] × [Pi], which involves complex energy transduction processes. The balance between ATP production and consumption is maintained by the mitochondrial oxidative phosphorylation capacity, which depends on metabolic substrates and the cell’s ability to effectively utilize the generated energy. In healthy hearts, ATP is produced mainly through fatty acid oxidation and, to a lesser extent, from glucose, lactic acid, ketone bodies, amino acids, etc. (Figure 2). Since the heart cannot store energy, it must produce it in a safe, efficient, and adequate manner.

In heart failure, metabolic remodeling occurs, diverting substrate utilization, reducing metabolic reserves, and leading to the production of harmful molecules (such as unfolded and heat shock proteins), increased thermal energy, Ca^2+^ overload, and altered mitochondrial and cytosolic function. This results in an abnormal distribution of ATP, ADP, Pi, and Pcr, ultimately leading to an inadequate energy supply (even though the total energy remains constant) and thereby worsening heart failure syndrome. Notably, energetic deficiency is observed in both the left and right ventricular myocardium, indicating a coordinated dysfunction across cardiac cells. However, the specific roles of each cardiac segment in this process are not yet fully understood [12].

The energetic status of each cell depends on metabolic remodeling regulated by energy sensors like AMP, which control, among other factors, phosphorylation states and ATP preservation [13]. These sensors also activate transcription factors and coactivators that influence long-term remodeling of ATP synthesis and utilization pathways [14,15,16]. Indeed, AMP-activated protein kinase (AMPK), peroxisome proliferator-activated receptor γ coactivator-1α (PGC-1α), the sirtuin family of proteins (SIRT), and peroxisome proliferator-activated receptors (PPARs)—all components of the AMPK/SIRT1/PGC-1α pathway—are crucial for the transcription of metabolism-related genes essential for maintaining mitochondrial energy homeostasis [17,18,19,20,21,22,23].

Beyond mitochondrial dysfunction, key pathways like AMPK and PGC-1α are central to preserving cardiac energy balance, especially under stress conditions such as heart failure (HF). AMPK functions as an energy sensor in cardiac cells, activating in response to low ATP levels. Once activated, AMPK stimulates pathways to increase ATP production and decrease ATP consumption by promoting glucose uptake, fatty acid oxidation, and mitochondrial biogenesis [24,25,26]. Notably, AMPK activation also enhances autophagy and inhibits protein synthesis, helping to conserve energy in HF [24,25,26]. PGC-1α, a coactivator of several nuclear receptors, drives mitochondrial biogenesis and enhances the oxidative phosphorylation capacity [25,26,27]. In HF, the downregulation of PGC-1α impairs mitochondrial biogenesis, decreasing ATP production and exacerbating energy deficiencies. Additionally, PGC-1α regulates antioxidant enzyme expression, and its dysregulation contributes to oxidative stress in cardiac cells [25,26,27]. Together, these pathways illustrate the intricate biochemical network that regulates energy metabolism in HF, providing potential targets for therapeutic intervention. Understanding how these signaling mechanisms integrate could offer insights into cardiac energy deficiencies beyond mitochondrial dynamics alone.

### Calcium Handling and Calcium/Calmodulin-Dependent Protein Kinase

Ca^2+^/calmodulin-dependent protein kinase (CaMKII) is present in cardiac myocytes and is activated by binding calcium/calmodulin complexes [28]. Upon binding four Ca^2+^ ions, calmodulin undergoes a conformational change, activating downstream proteins such as adenylate cyclase, serine/threonine kinases, nitric oxide synthase, and serine/threonine protein phosphatases, which are all involved in intracellular calcium signaling transduction [29]. Additionally, calmodulin can regulate calcium transport via plasma membrane ATPase [30], bind to several structural proteins essential for plasma membrane integrity and cytoskeletal structure [31], and influence significant cellular mechanisms, including cell cycle regulation, fertilization, intracellular signaling, differentiation, cell death, and contraction [32].

In the heart, CaMKII facilitates calcium entry into the cytoplasm and phosphorylates ryanodine receptors and phospholamban, thereby increasing the contractile force during a shorter cardiac cycle, which enhances ATP production through mitochondrial calcium entry [28,33]. CaMKII also supports diastolic relaxation by increasing Ca^2+^ reuptake and sequestration into the sarcoplasmic reticulum via SERCA activation [34]. In heart failure, reduced mitochondrial Ca^2+^ uptake leads to an altered NADH/NAD^+^ and FADH_2_/FAD^+^ oxidation state, which increases oxidative stress and reactive oxygen species (ROS) production [35].

Calcium plays a crucial role in maintaining cellular energy balance by activating mitochondrial dehydrogenases, enzymes essential for cellular respiration and energy production. Calcium ions entering the mitochondria activate key dehydrogenases, such as isocitrate dehydrogenase and α-ketoglutarate dehydrogenase, within the tricarboxylic acid (TCA) cycle [36,37,38]. This activation enhances the production of reducing equivalents—NADH and FADH_2_—that fuel the electron transport chain (ETC) and drive ATP synthesis [36,37,38]. This process is particularly vital in energy-demanding tissues like the myocardium, where a steady supply of reducing equivalents is crucial for sustaining cardiac output and function. Thus, calcium’s regulation of mitochondrial dehydrogenase activity directly supports cellular energy metabolism, linking calcium signaling to the maintenance of myocardial bioenergetics [36,37,38].

Conversely, an alternative hypothesis suggests that mitochondrial calcium overload activates mitochondrial permeability transition (MPT) pore opening, which dissipates the mitochondrial membrane gradient and releases pro-apoptotic factors, leading to cell death [39]. MPT pore opening is also closely associated with increased ROS production, significantly contributing to oxidative stress in the myocardium. Under conditions of mitochondrial calcium overload or energetic stress, MPT pore opening disrupts mitochondrial membrane potential, resulting in uncontrolled ROS generation [40,41,42]. This overproduction of ROS damages mitochondrial components and propagates oxidative stress within cardiomyocytes, activating pathways that lead to apoptosis and tissue remodeling [40,41,42]. This sequence exacerbates myocardial injury and contributes to the progression of heart failure, where excessive ROS play a pivotal role in mitochondrial dysfunction and cellular oxidative damage [40,41,42].

In heart failure, an increase in cytosolic calcium can result from excessive calcium influx or reduced calcium efflux from the cytosol [33]. Another pathophysiological change in heart failure is the rise in end-diastolic cytosolic calcium levels and prolongation of the calcium transient during diastole, due to reduced sarcoplasmic reticulum calcium uptake caused by decreased expression and activity of cardiac SR calcium ATPase (SERCA2a) [43]. CaMKII contributes to adverse remodeling through dysregulated calcium homeostasis and impaired calcium handling [44]. Targeted therapies aimed at enhancing mitochondrial Ca^2+^ uptake or preventing Ca^2+^ extrusion could be beneficial for heart failure patients [44]. Furthermore, CaMKII inhibition may offer an effective approach for treating heart failure and arrhythmias [45].

Finally, mitochondrial potassium channels are crucial in maintaining mitochondrial membrane potential and mitigating oxidative stress, particularly in cardiomyocytes. The ATP-sensitive potassium (mitoK-ATP) channel and large-conductance calcium-activated potassium (BK) channel help to regulate potassium flux into the mitochondria, stabilizing membrane potential under stress conditions [46,47,48,49]. In heart failure, the dysfunction of these channels compromises mitochondrial function, increasing the risk of mitochondrial depolarization and ROS overproduction [46,47,48,49]. Enhancing mitochondrial potassium channel function has shown cardioprotective benefits by restoring mitochondrial homeostasis, as demonstrated in both experimental and hereditary heart failure models [46,47,48,49]. Thus, modulating mitochondrial potassium channels presents a promising therapeutic strategy to reduce cardiac stress and improve myocardial resilience in heart failure.

## 3. Mitochondria, the Energetic Drive Machine

Mitochondria are intracellular organelles, often referred to as the “powerhouses of the cell”, responsible for maintaining efficient energy production to meet the energetic needs of each cell, tissue, and organ. The heart, being the most energetically active organ, contains more mitochondria than any other organ in the human body [50]. Mitochondria are located within the sarcolemma, are considered discrete entities, and must respond immediately to changes in energetic demand. Notably, mitochondrial dysfunction can impact the heart’s energetic status and contribute to various pathological conditions. Mitochondrial abnormalities directly affect cardiomyocyte injury and death, serve as a source of reactive oxygen species (ROS) production, and impair cardiac cell function. Furthermore, mitochondria are involved in cellular calcium homeostasis, vascular smooth muscle function, myofilament integrity, and cell differentiation [51].

ATP production is regulated by mitochondria through the control of fatty acid oxidation and oxidative phosphorylation. Mitochondrial dysfunction or abnormalities can reduce cardiac contractile capacity, impair Ca^2+^ flux turnover and homeostasis, promote the overactivation of inflammatory responses, and disrupt the genetically encoded regulation of cell death. As the “energy custodians” of the cell, mitochondrial homeostasis is essential and is supported through coordinated processes like mitochondrial biogenesis, mitophagy, and autophagy, along with the activation of specific proteins that recognize and remove damaged mitochondria [50,52,53]. Proper mitochondrial biosynthesis and degeneration are critical; imbalance in these processes reduces antioxidative capacity, initiates heart failure, and can lead to progressive disease. Biosynthesis and degeneration are complex functions that require precise biochemical regulation, including mitochondrial protein import, phospholipid synthesis, nuclear and mitochondrial protein control, mitochondrial fusion and fission, and replication of mitochondrial DNA. Disruption of these processes leads to dysregulation of the electron transport chain, reduced energy production, decreased available energy for cellular use, and initiates and exacerbates heart failure [50].

### 3.1. Mitochondrial Self-Protection

Mitochondrial homeostasis and function are primarily maintained through specific mechanisms: fission, fusion, mitophagy, and autophagy. Autophagy is a delicate process that selectively eliminates decaying mitochondria. Damaged mitochondria are renewed or removed through an intelligent turnover of fission and fusion processes, which facilitate their repair or removal through mitophagy and, ultimately, autophagy [54]. In heart failure, mitochondrial activities become unbalanced, with reduced fusion and increased fission activity [55], causing mitochondrial network instability. This imbalance, documented in both human and animal models and associated with several cardiac disease states, demonstrates a loss of mitochondrial homeostasis [56,57,58]. The processes of fission and fusion, collectively termed mitochondrial dynamics, are essential for clearing damaged mitochondria and eliminating specific damaged proteins. They facilitate the exchange of proteins and RNA between mitochondria, preventing the accumulation of harmful mtDNA mutations, misfolded protein aggregates, and excessive free radicals. Thus, proper mitochondrial dynamics are critical to maintaining energy efficiency and preventing the progression of heart failure [53].

Heart failure is associated with mitochondrial dysfunction, including abnormal mitochondrial structure, impaired ion homeostasis, increased ROS production, and diminished ATP generation [44]. Mitochondrial respiratory capacity and cardiolipin levels—an important phospholipid within the inner mitochondrial membrane—are observed to be reduced in heart failure [59]. Cardiolipin prevents cytochrome c release into the cytoplasm, a regulator of cellular apoptosis within the inner mitochondrial membrane. ROS-mediated oxidation and the depletion of cardiolipin activate caspases, leading to increased ROS production, cardiolipin peroxidation, mitochondrial dysfunction, and cellular death in the failing myocardium [60]. Abnormal mitochondrial energy metabolism in cardiac cells results in decreased ATP and excessive ROS production [61].

The sirtuin family (SIRT), a group of NAD^+^-dependent histone deacetylases, regulates multiple cellular pathways involved in various pathophysiological processes. Specifically, sirtuins inhibit inflammatory responses via the NF-κB pathway, reduce oxidative stress [62], and decrease TNF-α secretion [63]. Sirtuins also regulate systemic insulin sensitivity through effects on pancreatic secretion [64]. Nuclear SIRT1 and mitochondrial SIRT3 are the most significant members, showing beneficial effects on cardiac remodeling and improved cardiac and mitochondrial function in diabetic [65] and dilated cardiomyopathy [66]. SIRT1 regulates glucose metabolism through AMPK upregulation [67], while SIRT3 inhibits aerobic glycolysis by suppressing hypoxia-inducible factor 1α (HIF-1α) [68]. Additionally, SIRT6 reduces oxidative stress by promoting AMPK expression [69], while SIRT2 induces apoptosis by upregulating cleaved caspase 3 and Bax and downregulating the anti-apoptotic protein Bcl-2 [70]. Beyond benefits in cardiac hypertrophy, myocardial fibrosis, and atherosclerosis, sirtuins are useful for distinguishing heart failure phenotypes, particularly in distinguishing HFpEF from reduced or mildly reduced EF, based on SIRT1 activity in peripheral blood mononuclear cells [71]. SIRT1 levels, for example, are reduced in HFpEF patients and further diminished in those recently admitted for acute decompensated heart failure [72].

Another important molecule, phosphoglycerate mutase 5 (PGAM5), is a mitochondrial serine/threonine phosphatase that regulates mitochondrial dynamics and programmed cell death, playing a key role in maintaining mitochondrial function through the regulation of mitochondrial quality control mechanisms [73]. PGAM5 can increase mitochondrial biogenesis and autophagy in response to mitochondrial impairment in cardiomyocytes, while also inducing apoptosis when mitochondrial damage is severe [74].

Alterations in both glycolysis and mitochondrial oxidative metabolism in the failing heart result from transcriptional changes in enzymes involved in these pathways, as well as redox state changes (altered NAD^+^ and NADH levels) and metabolite signaling that contribute to epigenetic modifications in energy metabolic gene expression [75].

### 3.2. Heart Failure Drug–Mitochondria Interaction

Current heart failure guidelines [76] recommend the administration of four main classes of medications: (1) renin–angiotensin system inhibitors (angiotensin receptor-neprilysin inhibitors (ARNi), angiotensin-converting enzyme inhibitors (ACEi), or angiotensin II receptor blockers (ARB)); (2) beta-blockers; (3) mineralocorticoid receptor antagonists (MRAs); and (4) sodium-glucose cotransporter-2 inhibitors (SGLT2i). These drugs reduce morbidity and mortality by alleviating pressure and volume overload and suppressing neurohormonal and sympathetic overactivity. Additionally, ACE inhibitors [77,78] and beta-blockers [79,80] may reduce lipid peroxidation and oxidative stress, improving the rate of energy production and consumption and protecting against mitochondrial damage [81].

SGLT2 inhibitors, in particular, have shown beneficial effects on metabolism and mitochondrial function [82,83,84], likely by offering β-hydroxybutyrate as a substrate for oxidation, which improves mitochondrial energetics [85]. Thus, there appears to be an additive, mitochondria-targeted role for these medications, complementing their established benefits of reducing myocardial oxygen consumption (improving load conditions and lowering heart rate) and enhancing the utilization of energy substrates (fatty acids, glucose, ketone body oxidation), underscoring the significant interplay between mitochondrial function and cardiac energetic homeostasis. However, it is also essential to consider that some drugs can adversely affect mitochondrial function by inhibiting the electron transport system, β-oxidation of fatty acids, and the citric acid cycle and by altering mitochondrial permeability, mtDNA replication, and protein synthesis, thereby impacting mitochondrial dynamics and homeostasis [53,86].

## 4. Substrate-Metabolism Alteration/Energy Deficiency in Heart Diseases

As previously discussed, the heart utilizes multiple substrates for energy to perform its essential functions. Alterations in substrate metabolism have been shown to play a significant role in the development of several cardiac diseases [87,88].

In heart disease, the observed energy deficiency is, among other factors, due to a reduction in intracellular oxidative reactions. Glucose metabolism is upregulated, anaerobic glycolysis and lactate production are increased, fatty acid β-oxidation is decreased, and long-chain fatty acids accumulate within the mitochondria. Consequently, pyruvate dehydrogenase activity is inhibited, Ca^2+^ transport is altered—leading to excessive intracellular accumulation—lactic acid is elevated, pH is reduced, and ROS production increases. These mechanisms disrupt the homeostatic status of cardiac cells, leading to contractile dysfunction and worsening the heart failure syndrome across various clinical contexts.

### 4.1. Ischemic Heart Disease

In ischemic heart disease, there is a change in substrate utilization, an induced dysfunction in energy metabolism, mitochondrial dysfunction, and an alteration in the [ATP]/[ADP] × [Pi] equation, marking the onset and gradual progression of heart failure. It should be noted, however, that in ischemic heart disease, substrate-metabolism alterations and subsequent energy depletion are largely dependent on O_2_ delivery rates. At the early stages, cardiac energetic status remains unaffected; however, as the disease advances, signs of energy deficiency become apparent [89,90,91].

### 4.2. Heart Failure

In heart failure, the oxidation of fatty acids, glucose, and branched-chain amino acids is reduced, while glycolysis, lactate, and ketone body oxidation are increased. In the early to middle stages of heart failure, energy production from substrate metabolism remains unchanged. However, as the syndrome progresses, a shift from fatty acid to glucose metabolism occurs [92], initially compensating for the heart’s energetic needs. Simultaneously, mitochondrial dysfunction becomes evident, and along with increased oxidative stress [93] and Ca^2+^ homeostasis disruption [94], these changes lead to metabolic imbalances and insufficient ATP production, which accelerate disease progression [95,96]. Notably, as heart failure advances, glycolysis produces about 30% less ATP than under normal conditions [97]. Additionally, decreased fatty acid oxidation results in their accumulation within the heart, which is associated with the severity of heart failure [98].

In patients with heart failure, a paradoxical metabolism has been observed: those with dilated cardiomyopathy often show reduced fatty acid oxidation [99], while in patients with diabetes, insulin resistance, or obesity, fatty acid oxidation is increased [100,101]. The dysregulation of glucose transporters (GLUT1 increases, while GLUT4 decreases), along with decreased expression of monocarboxylate transporters and pyruvate dehydrogenase [102,103], suggests metabolic alterations and a decline in oxidative processes. This results in enhanced glycolysis, further lactate accumulation, mitochondrial dysfunction, and impaired myocardial function [104]. Ketone oxidation is increased [105], whereas branched-chain amino acid oxidation is reduced [106], which results in high plasma levels that may, although not fully proven, serve as an early indicator of cardiovascular disease [107].

D-dimer, a non-specific biomarker derived from the formation and degradation of cross-linked fibrin, reflects coagulation and fibrinolysis activation. Although primarily used to rule out pulmonary embolism in cases of low suspicion, some studies suggest its relevance in heart failure (HF). Specifically, in end-stage HF secondary to idiopathic dilated cardiomyopathy, elevated D-dimer levels are independently associated with poor long-term outcomes, including an increased risk of all-cause mortality (HR = 2.315, 95% CI: 1.570–3.414, *p* < 0.001) and major adverse cardiovascular events (MACE) (HR = 1.256, 95% CI: 1.058–1.490, *p* = 0.009), with a predictive value superior to other traditional markers [108]. In a recent study by de Boer RA et al. [109], elevated D-dimer levels were shown to predict incident systolic HF. Elevated D-dimer levels may also predict adverse outcomes in HF, such as a poor medium-term prognosis [110] and increased cardiovascular mortality [111]. Potential mechanisms underlying D-dimer’s prognostic use in HF include the following: (i) patients with elevated D-dimer often have a lower systolic blood pressure and left ventricular ejection fraction, indicating higher disease severity [112]; (ii) elevated D-dimer levels correlate with higher stroke and bleeding incidence [113]; (iii) D-dimer promotes inflammatory reactions by inducing cytokine release, increasing mortality risk [114]; and (iv) elevated D-dimer reflects hemostatic abnormalities, which may correlate with disease severity [112]. Finally, D-dimer could serve as a predictive marker of HF outcomes, as combining it with the GWTG-HF risk score and NT-proBNP enhances the early prediction of 12-month mortality in acute decompensated HF patients, regardless of phenotype [115].

ROS production regulates intracellular oxidative balance, with increased oxidative stress leading to cellular inflammation and programmed cell death [116]. Oxidative stress is driven by ROS overproduction, including free radicals and non-radical intermediates, and contributes to cardiovascular disease [117]. Impaired superoxide dismutase function, an enzyme responsible for ROS clearance, results in elevated baseline oxidative stress levels [118]. While mitochondria are the main source of endogenous ROS through electron transport chain by-products and oxidative phosphorylation [119], other enzymes outside mitochondria, such as nitric oxide synthase (NOS) and NADPH oxidase, also contribute to ROS production [120]. Additionally, environmental factors such as smoking, pollutants, ultraviolet radiation, xenobiotics, and alcohol increase exogenous ROS production through distinct mechanisms [121]. Intracellular ROS are highly reactive and unstable, modifying proteins, lipids, and nuclear structures, affecting protein function and signaling pathways [116]. ROS can lead to protein carbonylation and mitochondrial DNA damage [122,123]. Oxidative stress, due to ROS overproduction, is a major regulator of systemic inflammation [124]. In heart failure with a reduced ejection fraction (HFrEF), ROS are primarily produced by damaged cardiomyocytes, promoting remodeling through cell death and fibrosis [125], while in heart failure with a preserved ejection fraction (HFpEF), ROS are mainly generated by endothelial cells [126]. In both cases, mitochondria are the main ROS sources. Excessive ROS production damages mitochondria, increasing ROS levels further, while accelerating myocardial remodeling by activating various hypertrophic signaling kinases and transcription factors [127,128]. Patients with HF exhibit reduced expression of antioxidant genes and detoxifying enzymes in both ischemic and non-ischemic conditions, although CYP1B1 transcript levels appear elevated [129]. Antioxidant compounds, including vitamins A and C, omega-3 fatty acids, and novel experimental antioxidants targeting specific enzymes and pathways, may offer promising therapeutic strategies for HF in the future [116]. Current clinical medications, such as melatonin, PCSK9 inhibitors, carvedilol, and metformin, are also known for their antioxidant properties [116]. Although the roles of miRNAs (e.g., miRNA-210 and miRNA-1) and nanoparticles are still under investigation, they show promising results [116]. Proposed future approaches for anti-oxidative stress therapies in HF patients include increasing endogenous antioxidant capacity and boosting the expression/activity of antioxidant-producing enzymes [130].

### 4.3. Diabetic Cardiomyopathy

In diabetic cardiomyopathy, metabolic status is significantly altered, leading to an accumulation of fatty acids, while glucose—although abundant—exhibits reduced uptake and metabolism [131,132]. In this context, characterized by either an absence of insulin (type I) or impaired insulin activity (type II), patients demonstrate increased fatty acid metabolism, reduced glucose metabolism, decreased GLUT4 protein expression, elevated peroxisome proliferator-activated receptor alpha (PPARα) expression, and increased expression of CD36 and fatty acid-binding proteins, alongside reduced pyruvate dehydrogenase activity [133,134,135]. Notably, the low rates of fatty acid and glucose oxidation contribute to the accumulation of lipid intermediates and reactive oxygen species (ROS) [136], exacerbating mitochondrial dysfunction and activating additional signaling pathways that promote myocardial cell death via necrosis and apoptosis. Along with Ca^2+^ homeostasis disruption, these processes result in cardiac systolic dysfunction [137]. Consequently, this altered metabolic homeostasis causes cardiac energy deficits and ventricular dysfunction [138], marking the onset of heart failure syndrome [139].

### 4.4. Other Clinical Scenarios

Pulmonary and systemic hypertension are characterized by cardiac wall hypertrophy. Hypertrophy, leads to substrate metabolic status change, oxidative process reduction, electron transport dysfunction, and reduced ATP production [6,140,141,142]. Indeed, it has been reported that in cases of pulmonary hypertension, mitochondria function is altered—showing a reduction in glucose oxidation and an increase in glycolysis [142]. Accordingly, it has been reported in an experimental model that this is also true in cases of systemic arterial hypertension demonstrating a mitochondrial metabolic impairment [141]. 

Based on the above-mentioned knowledge, it is clear that the heart, under different circumstances and according to energetic needs, uses multiple energy substrates that in some cases can lead to heart metabolic remodeling [143]. Consequently, it seems that maladaptive metabolic remodeling [144,145] is associated with phenotypic alterations leading to structural and functional remodeling, having as a start point the energy production-consumption disequilibrium and the transition to heart failure [44]. Indeed, a reduction in the PCr/ATP ratio has been reported in various cardiac diseases, showing a correlation with a higher New York functional class, reduced left ventricular ejection fraction, and worse outcome [146].

## 5. Identifying Cardiac Energetic Status

Adequate cardiac mechanical efficiency depends on the use of aerobic oxidation of substrates, oxygen consumption, and consequently, the heart’s energetic status [147]. In this regard, it is valuable to identify ways to estimate oxidative metabolism, and thus, cardiac mechanical efficiency. It should be noted, however, that the calculated energy accounts for only 25% of consumed oxygen, with the remaining oxygen utilized in non-mechanical activities such as heat production, basal metabolism, and excitation–contraction coupling.

There are two primary approaches to estimating the mechanical energetic status: invasive and non-invasive methods. The invasive approach allows for the estimation of both input and output energy. Input energy can be measured using the Fick principle, by calculating coronary sinus blood flow multiplied by the arteriovenous oxygen content difference. Output energy, which is the sum of external work and potential energy generated by the heart per beat, can be calculated using the pressure–volume loop of the cardiac cycle. However, these invasive procedures are not easily applicable in everyday clinical practice.

The non-invasive method mainly relies on imaging techniques, including (a) positron emission tomography (PET), (b) cardiovascular magnetic resonance spectroscopy, both utilized in various clinical scenarios [148], and (c) metabolic disturbances identified in plasma [149]. Despite the emergence of artificial intelligence and nanotechnology in recent years, these imaging techniques are well established in clinical practice rather than novel.

In PET, commonly used tracers such as carbon-11–labeled acetate (^11^C-acetate) and oxygen-15–labeled molecular oxygen (^15^O_2_) have shown reasonable estimations of both myocardial blood flow and the oxygen extraction fraction, and hence myocardial oxygen consumption [147]. However, several drawbacks limit their widespread application. For instance, ^11^C-acetate provides only a semiquantitative index of oxidative metabolism, and although ^15^O_2_ is promising, its reliance on multiple tracers has restricted its use. Additionally, both techniques are costly, limiting their accessibility.

Phosphorus (31P) magnetic resonance spectroscopy (MRS) can measure both endogenous cardiac high-energy phosphate metabolites and creatine kinase (CK) flux in human hearts [150,151,152]. Creatine kinase is part of the principal equation [ATP]/[ADP] × [Pi] and functions as an energetic buffer between mitochondria and myocardial cells [6]. Consequently, a reduced PCr/ATP ratio may be a reliable marker of cardiomyocyte energy deficiency, as has been reported in various studies across different clinical scenarios [153,154,155]. Notably, CK flux at rest can be reduced by up to 65% [150,156] even when ATP levels are not significantly decreased.

Metabolic disturbances are common in patients with heart failure (HF). Plasma concentrations of metabolites such as histidine, phenylalanine, ornithine, and spermine, among others, can provide valuable insights into cardiac energetic status. Dysfunctional mitochondria in heart failure patients lead to abnormal utilization of biochemical substances and may contribute to the multi-organ functional failure associated with HF [149,157,158,159].

While metabolomics demonstrates significant diagnostic value in estimating HF-related metabolic disturbances [149], these changes indicate global rather than heart-specific alterations in metabolism. To address some of these limitations, an alternative method, proton spectroscopy (H-NMR), has been proposed, which has provided a detailed assessment of energetic profiles in mouse models, revealing significant alterations after just one minute of no-flow ischemia [160].

In any case, cardiovascular magnetic resonance spectroscopy can provide important information. However, its use is limited by lengthy scanning times, low specificity, and its limited capacity to study certain metabolic molecules adequately [161]. Nevertheless, it is worth noting that this technique has the potential to advance our understanding of the physiological and pathological aspects of cardiac metabolism. Importantly, it can examine metabolic changes in specific cardiac regions, providing a more comprehensive understanding and potentially uncovering regional differences in substrate utilization. By illuminating distinct metabolic alterations that may arise in specific heart regions (e.g., base, apex, walls, or layers), this approach may help in identifying early stages of the syndrome.

## 6. Medical Therapies Targeting Cardiac Metabolism

**Figure 3:**
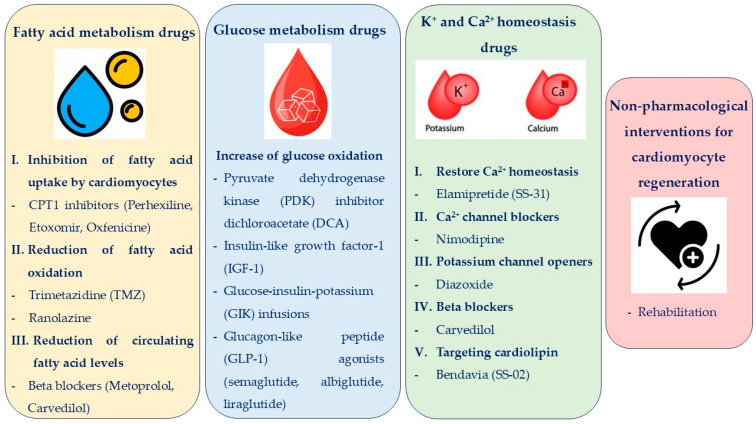
A brief illustration of medical therapies targeting cardiac metabolism and non-pharmacological interventions on cardiomyocyte regeneration.

During the last decades, new promising treatment strategies targeting the modulation of metabolic pathways have shown beneficial effects on clinical outcomes in patients with HF. The most significant pathways, which improve cardiac contractility by increased ATP availability and better fuel efficiency, include fatty acid and glucose metabolism. Specifically, the inhibition of fatty acid uptake by cardiomyocytes and the reduction in fatty acid oxidation and circulating fatty acids levels, as well as an increase in glucose oxidation, are the main therapeutic strategies targeting cardiac metabolism in HF [162]. A summarized view of medical therapies targeting cardiac metabolism is demonstrated in Figure 3.

### 6.1. Fatty Acid Metabolism Drugs

#### 6.1.1. Inhibition of Fatty Acid Uptake by Cardiomyocytes

Fatty acid oxidation is the primary source of ATP for the myocardium in healthy individuals, unlike in patients with heart failure (HF), where a reduction in mitochondrial fatty acid oxidation is observed. This decline in fatty acid oxidation is associated with the progression of HF syndrome [163,164,165]. Consequently, a 25–30% reduction in ATP levels is inevitable in the failing heart [166]. Studies have shown promising results for medical therapies targeting fatty acid metabolism. These drugs aim to balance myocardial fatty acid uptake and oxidation by reducing circulating fatty acids to mitigate lipotoxicity and enhance bioenergetics.

The first therapeutic approach targeting fatty acid metabolism in HF focuses on inhibiting fatty acid uptake by cardiomyocytes. Carnitine palmitoyltransferase 1 (CPT1) is an enzyme regulating fatty acid entry into mitochondria; thus, CPT1 inhibitors, including perhexiline and etomoxir, may offer potential therapeutic benefits [162]. CPT1 inhibitors decrease this enzyme’s activity, thereby limiting fatty acid oxidation and enhancing glucose oxidation [167]. Perhexiline, a specific cardiac isoform of the CPT1 inhibitor, is well studied in HF. Initially used as an anti-anginal agent, perhexiline has shown beneficial effects on exercise capacity and clinical outcomes in HF patients. Specifically, it has been found to improve the peak VO_2_ [168,169], left ventricular ejection fraction (LVEF) [169], and myocardial energetics [168,170] in HF patients.

In a 2005 study, Lee et al. [169] examined the effects of perhexiline in 56 HF patients. This therapy appeared to relieve symptoms and improve the peak VO_2_ by 17%, LVEF by 42%, and the quality of life. It also enhanced resting dobutamine stress regional myocardial function by 15% and peak dobutamine stress regional myocardial function by 24%, along with skeletal muscle energetics by normalizing phosphocreatine recovery after exercise [169]. In a large retrospective UK study of 151 patients with chronic HF and/or refractory angina [171], perhexiline therapy provided symptomatic relief for most patients, with minimal side effects or toxicity. Another study by Beadle et al. [170] showed that short-term perhexiline therapy improved the New York Heart Association (NYHA) functional class and cardiac energetics by increasing the myocardial phosphocreatine/adenosine triphosphate (PCr/ATP) ratio by 30%, without a shift in substrate utilization in patients with systolic HF of nonischemic etiology. However, no statistical improvements were seen in LVEF or respiratory exchange ratio [170]. These findings align with a prior study by the same researchers, which showed that a 5-month perhexiline therapy improved the PCr/ATP ratio, diastolic dysfunction, and exercise capacity, as measured by peak VO_2_, in patients with hypertrophic cardiomyopathy [168].

The effects of perhexiline in patients with aortic stenosis are controversial. An early study by Unger et al. in 1997 [172] showed clinical improvement in 13 of 15 elderly patients with severe aortic stenosis, with 5 becoming asymptomatic after a 30-month follow-up. However, findings from a more recent large multicenter, double-blind, randomized controlled trial involving 112 patients (54 perhexiline, 48 placebo) with left ventricular hypertrophy secondary to aortic stenosis undergoing aortic valve replacement were less promising [173]. Perhexiline did not offer additional benefits in hemodynamic performance or attenuate myocardial injury, as there was no difference in inotrope usage, myocardial injury (as assessed by ECG), or postoperative troponin release between groups. A notable limitation was that nearly 40% of patients fell below the therapeutic range of serum perhexiline concentration.

In summary, perhexiline inhibits fatty acid uptake by cardiomyocytes and may be promising for HF treatment, in addition to its known pharmacological effects, such as L-type calcium channel inhibition, inhibition of HERG and Kv1.5 potassium channels, potentiation of insulin release, and reductions in platelet reactivity mediated by cyclic guanosine monophosphate and nitric oxide [174]. Ongoing phase 3 clinical trials, such as the RESOLVE-HCM trial, are examining the effects of perhexiline on the extent of left ventricular hypertrophy (LVH) using cardiovascular magnetic resonance (CMR) in symptomatic hypertrophic cardiomyopathy (HCM) patients with moderate-to-severe LVH [175].

On the other hand, etomoxir is less extensively studied but shows promising results in HF patients. Originally developed for diabetes mellitus treatment, etomoxir, a CPT1 inhibitor, was shown to improve clinical status, central hemodynamics (maximum cardiac output and stroke volume) at rest and during exercise, and LVEF in a small clinical trial of 10 patients with chronic congestive HF, though it exhibited neither positive inotropic effects nor vasodilatory properties [176]. Etomoxir also showed beneficial effects on functional capacity in pressure-overloaded left ventricles and myocardial performance [177] and delayed HF progression in pressure-overload cardiac hypertrophy by enhancing sarcoplasmic reticulum uptake in animal studies involving rats [178]. However, etomoxir’s potential neurotoxicity and hepatotoxicity are significant limitations [167]. Interestingly, oxfenicine, another CPT1 inhibitor, was tested in an animal model and found to prevent left ventricular wall thinning and delayed progression to end-stage failure when administered early in HF in dogs [179].

#### 6.1.2. Reduction in Fatty Acid Oxidation

The second therapeutic approach for cardiac metabolism in HF involves reducing fatty acid oxidation. Medical therapies often aim to reduce fatty acid oxidation by targeting mitochondrial enzymes that contribute to the β-oxidation of fatty acids [167]. These therapies include trimetazidine (TMZ) and ranolazine. TMZ inhibits 3-ketoacyl coenzyme A thiolase, a key enzyme in fatty acid oxidation [167]. TMZ has demonstrated beneficial effects by decreasing free fatty acid oxidation, inhibiting oxidative phosphorylation, and improving glucose utilization and ATP production. Furthermore, it has been associated with improvements in cardiac function and functional class [180,181,182,183,184,185], exercise capacity [185], and LVEF [180,183,184,186] in HF patients. TMZ preserves intracellular phosphocreatine and ATP levels, reduces cellular acidosis and free radical-induced injury, and shifts energy production from free fatty acid to glucose oxidation by inhibiting oxidative phosphorylation [187]. Additionally, it may provide cardiac protection by inhibiting fibrosis, ischemia/reperfusion injury, cardiomyocyte apoptosis, and oxidative stress through pathways such as the NADPH oxidase/ROS/connective tissue growth factor pathway [188], AMP pathways [189], and the mitochondrial pathway [190].

In 2003, Fragasso et al. [181] demonstrated that TMZ increased LVEF, improved symptom relief, NYHA class, glucose metabolism by lowering fasting blood glucose, and endothelial function by reducing endothelin-1 in 16 patients with diabetes and ischemic hypokinetic cardiomyopathy compared to controls. Subsequently, the same investigators confirmed these findings, showing that the addition of TMZ improved exercise tolerance and duration and decreased brain natriuretic peptide (BNP) levels compared to conventional therapy alone, though without significant improvements in quality of life [180]. Importantly, TMZ was equally effective in both ischemic and nonischemic HF [180]. In subsequent studies, TMZ was shown to beneficially affect whole-body resting energy expenditure (REE), related to increased serum fatty acid oxidation. Both REE and serum fatty acid oxidation correlate inversely with LVEF and positively with increased whole-body oxygen consumption and circulating fatty acids [182]. Regarding other echocardiographic indices, TMZ improved both LV and RV myocardial velocities, suggesting benefits in RV function, especially in patients with diabetes, except in cases of ischemic HF alone [186].

In addition to clinical trials, meta-analyses have confirmed TMZ’s beneficial effects on cardiac function. The first meta-analysis, comprising 17 studies with 955 HF patients, found a significant association between TMZ and increased exercise duration, LVEF in both ischemic and non-ischemic HF, reduced LV end-systolic volume, and improved NYHA classification, along with significant reductions in all-cause mortality, cardiovascular events, and hospitalizations [191]. A more recent meta-analysis by Zhao et al. [192], which included six studies with 310 non-ischemic HF patients, emphasized TMZ’s benefits on exercise endurance and cardiac function, showing improvements in 6-MWT, LVEF at 3 and 6 months, and peak oxygen consumption. The latest meta-analysis by Nassiri et al., in 2024 [193], associated TMZ with reduced cardiovascular mortality and HF hospitalizations, as well as improvements in the NYHA class, 6MWT, and quality of life in 2552 patients with both ischemic and non-ischemic cardiomyopathy and reduced EF across 28 studies. The significant clinical impact of TMZ in more severe HF cases may stem from the notable differences in myocardial fuel flexibility between HFrEF and HFpEF [194].

The benefits of TMZ extend beyond cardiac function and echocardiographic indices. Patients with dilated ischemic cardiomyopathy treated with TMZ displayed an improved inflammatory profile, marked by lower plasma C-reactive protein concentrations compared to those not receiving TMZ [183]. Higher C-reactive protein levels in HF are known to correlate with increased hospital readmission and mortality rates [195]. Additionally, TMZ appears to reduce NT-proBNP levels and improve exercise tolerance in diabetic patients with idiopathic dilated cardiomyopathy after a six-month follow-up [185]. Intriguingly, TMZ influences electrophysiological pathways, potentially useful for managing major arrhythmias. Specifically, Cera et al. evaluated TMZ’s effect on atrial depolarization and ventricular repolarization in 30 postischemic HF patients [196]. They observed a significant reduction in the Tpeak–Tend-d index, a noninvasive marker of ventricular repolarization dispersion, in patients treated with TMZ compared to controls, suggesting a reduced morbidity and mortality risk from life-threatening arrhythmias in this patient subgroup. Although the pathophysiology remains unclear, TMZ’s antiarrhythmic effects may be mediated by its anti-ischemic properties [196]. Finally, Tuunanen et al. [184] demonstrated a synergistic effect of TMZ on both cardiac and extracardiac metabolic processes in patients with idiopathic dilated cardiomyopathy, significantly reducing insulin resistance, increasing plasma HDL by 11%, and decreasing cardiac fatty acid oxidation by 10%, with myocardial oxidative rate unchanged. These findings imply that TMZ may mitigate myocardial damage associated with insulin resistance in HF [184].

In contrast, some clinical trials have not shown improvements in specific aspects of cardiac function or exercise capacity. For instance, Bohdan et al. observed no changes in peak VO_2_ uptake, 6MWT, LVEF, quality of life, mortality, or cardiovascular events in 45 patients with stable advanced HFrEF compared to placebo [197]. Similarly, the DoPING-HFpEF trial found no benefits of TMZ over placebo for pulmonary capillary wedge pressure at various exercise levels, myocardial PCr/ATP ratio, 6MWT, NT-proBNP, quality of life, echocardiographic or CMR measures of diastolic function, or metabolic parameters in 25 HFpEF patients [198]. In summary, TMZ offers a favorable pharmacological profile with relatively low cost and occasional adverse reactions, suggesting potential for broader use in HF management in the future, although further randomized clinical trials are warranted.

Ranolazine, an inhibitor of the cardiac late sodium current (INaL), also impacts fatty acid metabolism. INaL elevation in HF leads to sodium (Na^+^) overload, which promotes an increased exchange of intracellular Na+ for extracellular Ca^2^+, causing Ca^2^+ overload and prolongation of the action potential [199]. Moreover, ranolazine is a partial fatty acid oxidation inhibitor that shifts cardiac energy metabolism from fatty acid oxidation to glucose oxidation, supporting myocardial function during ischemia and reducing ROS formation [200]. Ranolazine’s beneficial effects in HF have been demonstrated in clinical trials. For instance, the RALI-DHF study [201] evaluated ranolazine’s effects in 20 HFpEF patients (12 on ranolazine and 8 on placebo), finding significant improvements in hemodynamic parameters like pulmonary capillary wedge pressure and mean pulmonary artery pressure, although there was no improvement in echocardiographic relaxation indices (tau or LV pressure decline rate), E/E′ ratio, cardiopulmonary exercise test parameters, or NT-proBNP. Later, the RE-STYLE-HCM randomized, double-blind study [202] examined ranolazine’s effects on exercise performance, plasma BNP levels, diastolic function, and quality of life in 80 patients with nonobstructive symptomatic hypertrophic cardiomyopathy, without finding beneficial effects over placebo. However, ranolazine did reduce the 24-h burden of premature ventricular complexes [202]. Subsequently, the ARETHA AT study [203] demonstrated that ranolazine was effective, safe, and well tolerated in 292 patients with stable angina, improving symptoms, reducing angina episodes and nitrate use, and enhancing quality of life. Another recent study, the RANGER study [204], assessed ranolazine’s efficacy and safety in 1,101 patients with stable angina and/or HF, confirming findings from the ARETHA AT study and indicating that ranolazine is safe, effective, and well tolerated. A recent meta-analysis of eight studies in HFpEF patients associated ranolazine with improved diastolic performance without affecting the blood pressure, heart rate, or ventricular repolarization rate [205].

#### 6.1.3. Reduction in Circulating Fatty Acid Levels

The third therapeutic approach of cardiac metabolism to indirectly balance fatty acid oxidation in HF is to reduce their circulating levels. High levels of circulating fatty acids lead to decreased glucose and pyruvate oxidation [206]. Decreasing fatty acid concentrations or directly inhibiting their oxidation increases pyruvate oxidation and therefore cardiac efficiency [207]. β-blockers including metoprolol and carvedilol as described above decrease myocardial fatty acid use and increase carbohydrate oxidation [208,209]. Moreover, they present various benefits in cardiac performance and survival in patients with HF [208,210]. 

### 6.2. Glucose Metabolism Drugs

The final significant aspect of cardiac metabolism in heart failure (HF) is the increase in glucose oxidation. Increased glucose oxidation is proposed to improve myocardial efficiency by generating more ATP per molecule of oxygen consumed in the failing heart [211]. In HF pathophysiology, deranged substrate metabolism, insulin resistance, and myocardial energetic deficiency are observed [212,213]. The pyruvate dehydrogenase kinase (PDK) inhibitor dichloroacetate (DCA), a pyruvate analog, increases PDK function and glucose oxidation. Data on DCA use in HF are limited due to concerns about chronic neurotoxicity [214]. Earlier studies showed no benefit in left ventricular (LV) function or exercise capacity in patients with congestive HF [215,216]. However, a single study by Bersin B et al., in 1994 [217], reported that DCA stimulated myocardial lactate consumption and improved LV mechanical efficiency, showing a significant increase in stroke volume and LV minute volume alongside reduced myocardial oxygen consumption in HF patients. Conversely, studies in animal models have demonstrated promising results. Specifically, a recent study by Li X et al. [218] showed metabolic modulation and cardioprotective effects of DCA in ischemic mouse hearts. Other studies in porcine HFpEF models [219] and in rats with compensated LV hypertrophy and HF [220] demonstrated improvements in myocardial contractility, reduced hypertrophy, improved survival, increased energy reserves, and reduced oxidative stress following DCA treatment.

Insulin-like growth factor-1 (IGF-1) has been proposed to enhance mitochondrial energy production through a Ca^2+^-dependent mechanism that supports oxidative metabolism during the adaptive growth of cardiomyocytes [221]. IGF-1 stimulation increases mitochondrial Ca^2+^ uptake in ventricular myocytes in animal models and in human embryonic stem cell-derived cardiomyocytes, indirectly reducing pyruvate dehydrogenase phosphorylation [221]. IGF1 receptor (IGF1R) activation in chronic HF is associated with improved cardiac contractility, physiological hypertrophy [222], and reduced apoptosis in myocytes subjected to ischemic injury [223]. IGF1R signaling and cardiac function exhibit a biphasic, age-dependent relationship rather than a linear one [224]. Additionally, IGF-1 promotes cardiac recovery after myocardial infarction by increasing cellular resistance to oxidative stress [225].

Glucose–insulin–potassium (GIK) infusions blunt the rise of IGFBP-1, thereby increasing IGF-1 bioavailability and accelerating its return to baseline—crucial for high-risk catabolic patients with low IGF-1 levels [226]. Accordingly, GIK infusions positively affect the GH/IGF-1 axis and have been used in HF, showing cardiac benefits, such as improved LV systolic function and decreased BNP levels in HFrEF patients [227]. They also enhance hemodynamics by raising the mean arterial pressure and reducing heart rate in septic shock patients with myocardial depression [228]. Additionally, GIK administration reduces the incidence of in-hospital major adverse cardiac events in HF patients undergoing cardiopulmonary bypass surgery [229]. Mechanistically, insulin appears to suppress inflammation and free fatty acids while enhancing glucose utilization, providing a more efficient energy source [230]. GIK may also improve Ca^2+^ homeostasis and thereby increase myocardial contractility in the stunned myocardium in HF [227]. It is generally well-tolerated, with minimal adverse effects in most patients [228].

Another pharmacological agent, glucagon-like peptide-1 (GLP-1) receptor agonists, aims to increase insulin secretion, sensitivity, and glucose uptake. Its activity is enhanced by IGF-1 receptor expression and secretion stimulation, a mechanism crucial for GLP-1-induced apoptosis protection [231]. Initially developed as diabetes medications, GLP-1 receptor agonists lower blood glucose and improve glucose metabolism via GLP-1 receptor activation [232]. All GLP-1 receptor agonists are administered subcutaneously, except for oral semaglutide. Numerous randomized controlled trials have explored the effects of GLP-1 agonists in HF over the past decade. Among these, only the HARMONY trial [233] demonstrated a significantly reduced hazard ratio for HF hospitalization and major adverse cardiovascular events with albiglutide compared to placebo. The subsequent REWIND trial [234] showed improvement in primary composite outcomes in HF patients receiving dulaglutide compared to placebo but did not observe differences in HF hospitalizations. Both the LEADER trial (assessing liraglutide) [235] and the SUSTAIN-6 trial (assessing semaglutide) [236] showed significant reductions in cardiovascular death, nonfatal myocardial infarction, or nonfatal stroke rates in high-risk patients with type 2 diabetes. A large meta-analysis of cardiovascular outcome trials [237] further showed the superiority of GLP-1 receptor agonists in cardiovascular, mortality, and kidney outcomes in patients with type 2 diabetes compared to placebo. However, other randomized controlled trials did not show significant benefits of lixisenatide [238], exenatide [239], or oral semaglutide over placebo for HF hospitalization and major cardiovascular events in HF patients. Collectively, a meta-analysis of these trials—including 60,080 patients with diabetes and cardiovascular disease—indicates that GLP-1 agonists reduce HF hospitalizations by 10–11% [240,241]. It remains unclear whether the benefit is greater in HFpEF or HFrEF. However, two studies assessing GLP-1 in HFrEF reported similar primary endpoint and HF hospitalization outcomes between the intervention and placebo groups [242,243]. Indeed, a recent meta-analysis of the FIGHT and EXSCEL studies [244] suggested that GLP-1 may increase the risk of hospitalizations in HFrEF. In conclusion, GLP-1 receptor agonists may be particularly beneficial for HF patients, especially those with established atherosclerotic cardiovascular disease and preserved EF, as they offer significant benefits and are generally well tolerated, with mainly minor gastrointestinal side effects such as nausea, vomiting, and diarrhea [245].

### 6.3. Approaches Improving Mitochondrial Calcium and Potassium Homeostasis

Strategies to restore Ca^2+^ homeostasis include the modulation of proteins such as those in the Ca^2+^–calmodulin kinase II (CaMKII) pathway, which mediates Ca^2+^ influx and regulates contractile force in cardiomyocytes by facilitating Ca^2+^ release and uptake in the sarcoplasmic reticulum [246,247,248]. CaMKII inhibition, therefore, is a potential therapeutic target for improving Ca^2+^ cycling efficiency and reducing oxidative stress. Additionally, K^+^ ion channels, which help to regulate mitochondrial membrane potential and reactive oxygen species (ROS) generation, present another target. By stabilizing K^+^ influx, mitochondrial health is preserved, thereby preventing excessive ROS production that would otherwise damage mitochondrial DNA and proteins [246,247,248]. This approach has shown particular effectiveness in models of diabetic cardiomyopathy and muscular dystrophy, conditions where mitochondrial dynamics and energetic efficiency are frequently compromised. Interventions that regulate mitochondrial Ca^2+^ and K^+^ thus have the potential to enhance cellular resilience in HF by reducing apoptosis, promoting efficient ATP synthesis, and minimizing oxidative damage across various cardiac pathologies [246,247,248].

Elamipretide (SS-31) is a mitochondrial-targeting peptide that stabilizes cardiolipin, a lipid essential for maintaining mitochondrial membrane structure and function. By preserving cardiolipin, elamipretide helps to optimize Ca^2+^ and K^+^ flux across the mitochondrial membrane, thereby supporting ATP production and reducing ROS formation [246,247,248]. Studies indicate that elamipretide improves mitochondrial efficiency, reduces Ca^2+^-induced mitochondrial dysfunction, and protects cells from oxidative stress, especially in models of HF and diabetic cardiomyopathy [249]. Nimodipine, a Ca^2+^ channel blocker of the dihydropyridine type, reduces Ca^2+^ influx in various cell types, including cardiac cells. Although commonly used for cerebrovascular conditions, it has shown potential in protecting mitochondrial function in cardiac cells by preventing excessive Ca^2+^ loading, which would otherwise trigger mitochondrial permeability transition pore (mPTP) opening. By limiting Ca^2+^ entry, nimodipine helps to maintain mitochondrial stability, supports ATP production, and reduces ROS generation [250].

Mitochondrial potassium channel openers such as diazoxide open mitochondrial ATP-sensitive K^+^ (mitoKATP) channels, facilitating K^+^ influx, stabilizing mitochondrial membrane potential, reducing excessive ROS production, and providing protection under stress [251]. In preclinical studies, diazoxide has shown benefits in ischemic heart disease by reducing mitochondrial stress and preserving cardiac tissue through ROS overload prevention and cellular injury mitigation [252]. Beta-blockers, including carvedilol—a non-selective beta-blocker with antioxidant properties—indirectly support mitochondrial Ca^2+^ and K^+^ homeostasis and reduce Ca^2+^ overload by modulating beta-adrenergic signaling, thereby decreasing Ca^2+^ entry and subsequent mitochondrial stress [81]. Carvedilol has been shown to enhance mitochondrial function by reducing Ca^2+^-induced oxidative stress, increasing mitochondrial stability, and improving energy efficiency in patients with HF and hypertension [253,254].

Finally, Bendavia (SS-02), another cardiolipin-targeting agent, stabilizes mitochondrial membranes and supports Ca^2+^ and K^+^ balance within mitochondria. It has demonstrated protective effects in models of ischemic heart failure and diabetic cardiomyopathy by reducing Ca^2+^-induced mPTP opening, improving ATP synthesis, and limiting ROS formation [255,256].

### 6.4. Non-Pharmacological Interventions on Cardiomyocyte Regeneration

Cardiac remodeling after ischemic injury results in cardiomyocyte loss, fibrosis, and therefore, impaired heart function [257]. Cardiomyocytes regeneration is limited in adults, and no direct clinical therapies for ischemic injury currently exist. The only direct therapeutic strategies in humans are revascularization in the acute phase and cell-based interventions including adult stem cells derived from bone marrow and adipose cells, cardiosphere-derived cells, skeletal myoblasts, and pluripotent stem cells, which are still under investigation. There are also molecular signals including growth factors, intrinsic signaling pathways, microRNAs, and cell cycle regulators that stimulate cardiomyocyte proliferation in HF [258]. Other indirect interventions for cardiomyocytes’ regeneration include left ventricle support device therapy, but this still remains a difficult operative procedure with complications for the patient [259].

Except for pharmacological therapies, non-pharmacological interventions are also quite significant for reversing cardiomyocyte damage in HF. Cardiac rehabilitation is the most important with the most promising results among them. Characteristically, a cardiac rehabilitation program based on aerobic and/or muscle strength training has been shown to stimulate the long-term mobilization of endothelial progenitor cells (EPCs) [260] and improve the nitric oxide synthase system and antioxidant system in patients with chronic HF [261]. Moreover, acute exercise has similar beneficial effects on the acute mobilization of EPCs in patients with chronic HF [262]. Indeed, the beneficial effects of exercise seem to be similar in HF patients of different severity [263]. The HF high inflammatory profile causing skeletal muscle wasting and dysfunction and the endothelial dysfunction leading to impaired endothelium dependent vasodilatation and impaired cardiac function both contribute to the progression of the disease and the deterioration of exercise intolerance [260]. It is well known that EPCs are being used as an index of the endothelium restoration potential, reflecting vascular endothelial function [264]. Exercise training has the privilege to increase blood flow, shear stress, and the levels of serum nitric oxide (NO), nitric oxide synthase (NOS), and superoxide dismutase (SOD) [261], therefore increasing the exercise capacity, stimulating EPCs, and reducing inflammation (interleukin-6 and CRP) [260,263]. Exercise-induced shear stress and ischemic/hypoxic stimulus are the suggested mechanisms behind the mobilization of EPCs [260]. EPCs are associated with cardiac function and cardiac metabolism as they hold a high potential to ameliorate atherosclerotic pathogenesis and restore endothelial dysfunction in HF, as well as promoting endothelial regeneration and neovascularization [263]. Moreover, cardiac rehabilitation is reducing LV end-diastolic and -systolic volumes in post-MI patients with preserved LVEF, indicating improvements in reverse LV remodeling [265]. Therefore, cardiac rehabilitation is an established therapeutic strategy in HF, along with medication, and physicians should prioritize its immediate incorporation as part of the standard of care for these patients. 

## 7. The Future

Although the pivotal role of energy production and utilization is well established, only a limited number of scientific reports have been published on this topic. Therefore, it is crucial to expand research in this field to gain deeper insights into the pathophysiological diagnosis of heart failure, the contribution of cardiac segments, and ultimately, therapeutic approaches for heart failure. Advanced diagnostic tools, including imaging techniques, metabolomics, proteomics, and artificial intelligence, offer valuable opportunities to explore the complex pathophysiological mechanisms at play.

Furthermore, considering that oxidative phosphorylation in mitochondria serves as the primary source of cardiac energy, attention must be directed toward mitochondrial function and dysfunction [266,267,268]. Several studies utilizing various microRNAs have demonstrated improvements in cardiac function, highlighting their potential as therapeutic targets [269,270,271]. Similarly, the use of mitochondrial reactive oxygen species (mtROS) scavengers, coenzyme Q10, and CGP 37175—a Na^+^/Ca^2+^ exchanger inhibitor—has shown promise in ameliorating mitochondrial remodeling and cardiac dysfunction [272,273,274], pointing to a novel therapeutic pathway.

In this context, more intensive efforts are necessary to fully understand the complexities of heart failure syndrome and thereby enhance medical treatment options.

### 7.1. Specialized Imaging Techniques and Biomedicine Branch

Novel and more specialized imaging techniques are being explored in order to offer unprecedented insights into the metabolic and structural abnormalities underlying HF. Specifically, positron emission tomography (PET), especially with metabolic tracers such as ^18^F-fluorodeoxyglucose (FDG), allows for the visualization of glucose uptake and utilization in the myocardium [275]. Recent advancements include the use of tracers like ^11^C-acetate, which provides information on oxidative metabolism, and 18F-fluorodopamine, which measures sympathetic nervous system activity. This detailed metabolic mapping helps in understanding how energy production is compromised in heart failure, particularly by examining how the heart switches from fatty acid oxidation to glucose metabolism under stress [275]. Another imaging technique, magnetic resonance imaging (MRI) with Phosphorus-31 spectroscopy (^31^P-MRS) remains a gold standard for visualizing heart structure and function, but its combination with ^31^P-MRS has been a game-changer for metabolic imaging in HF [151]. Phosphorus-31 spectroscopy allows for the non-invasive measurement of high-energy phosphate compounds, including ATP and PCr. These are critical to understanding the bioenergetic status of the heart [151]. A reduced PCr/ATP ratio is a hallmark of HF, reflecting the impaired energy supply necessary for contraction. Single photon emission computed tomography (SPECT) is similar to PET but more widely available and affordable. New developments in SPECT imaging include the use of technetium-99m sestamibi to assess myocardial perfusion and iodine-123 metaiodobenzylguanidine (^123^I-MIBG) for evaluating sympathetic nerve activity [276]. These advances help clinicians to understand how alterations in blood flow and nerve signaling contribute to the energy deficit seen in HF [276]. Magnetic resonance spectroscopy (MRS) with Carbon-13 (^13^C-MRS) allows real-time tracking of substrate utilization in the myocardium [277]. By using 13C-labeled substrates, such as glucose or fatty acids, crucial insights into the metabolic shifts that occur in HF are provided, enabling researchers to map changes in real time and better understand how therapeutic interventions might restore normal energy utilization [277]. Finally, dynamic nuclear polarization (DNP)-enhanced MRI dramatically increases the sensitivity of MRI in detecting metabolic changes [278]. DNP-enhanced MRI uses hyperpolarized molecules to trace the metabolism of key energy substrates in the heart, such as pyruvate, in real time, providing detailed information on cellular bioenergetics and offering the potential to identify metabolic shifts in HF much earlier than conventional imaging methods [278].

The branch of biomedicine is defined as a wide range of scientific disciplines and specialized fields that focus on understanding biological processes and their applications in medicine. This branch integrates various areas of expertise, often identified by the “bio-” prefix, such as molecular biology, biochemistry, biotechnology, cell biology, embryology, nanobiotechnology, bioengineering, medical laboratory sciences, cytogenetics, genetics, gene therapy, bioinformatics, etc. [279]. These fields work together to explore the molecular and cellular mechanisms underlying health and disease, develop innovative diagnostic and therapeutic tools, and improve medical interventions to enhance patient care [279]. It plays a crucial role in advancing our understanding of HF, developing innovative therapies, and improving patient outcomes. By integrating knowledge from various scientific disciplines, biomedicine facilitates a comprehensive approach to tackling the challenges posed by HF, ultimately contributing to enhanced healthcare and improved quality of life for affected individuals. Molecular biology and biochemistry help to elucidate how alterations in metabolic pathways, gene expression, and protein function contribute to the development and progression of heart failure. The field of genetics and genomics has significantly advanced our knowledge of the hereditary aspects of HF, driving the development of new therapeutic strategies as gene therapy and cell therapy. Moreover, the identification of novel biomarkers through bioinformatics and proteomics has improved the diagnosis and management of HF. 

### 7.2. Nanotechnology

Nanotechnology has enabled the development of molecular imaging agents that target specific molecular pathways involved in HF. Nanoparticles can be engineered to bind to key markers of inflammation, fibrosis, or apoptosis in the myocardium, allowing these processes to be visualized with high precision [280,281]. This opens the door for more accurate detection of early pathological changes at the cellular level before they manifest as functional decline. Nanotechnology could offer innovative approaches that improve patient outcomes. In terms of diagnostics, nanobiosensors and nanomaterials like In_2_O_3_ nanoribbons and carbon nanotubes are being developed to detect biomarkers such as troponin and BNP with high sensitivity, enabling the early identification of HF [280,281]. Moreover, the use of nanomaterials such as liposomes, polymers, and inorganic nanoparticles is improving the sensitivity and specificity of diagnostics through biosensors and molecular imaging techniques like MRI, optical imaging, and nuclear scintigraphy [281]. As far as treatment is concerned, nanoparticles are used for targeted drug delivery, allowing medications to be more precisely directed to the heart while reducing systemic toxicity. Polymeric nanocarriers improve the pharmacokinetics and pharmacodynamics of drugs, enhancing myocardial contractility and reducing adverse effects [280,281]. Additionally, magnetic and gold nanoparticles, as well as collagen hydrogels, are being tested for their ability to regenerate damaged heart tissue by promoting cardiomyocyte growth and improving heart muscle function [280,281]. Specifically, nanomaterials have a significant role in targeted drug delivery by reducing plaque formation in atherosclerosis and providing diagnostic insights into the disease’s progression [281]. These nanotechnologies not only improve early detection and targeted therapy but also open possibilities for tissue regeneration and functional recovery in HF patients.

### 7.3. Artificial Intelligence

Artificial intelligence (AI)-powered algorithms are revolutionizing the analysis of imaging data, providing enhanced accuracy and earlier detection of cardiac abnormalities in function and structure. Machine-learning models can analyze large imaging datasets to identify subtle patterns or abnormalities that may signal early HF. AI can also predict how the disease will progress, personalize treatment plans, and identify the best time for therapeutic interventions [282]. AI is increasingly used in both the diagnosis and treatment of HF. In diagnosis, AI algorithms are applied to imaging technologies like echocardiography, CMR, and cardiac CT to automate assessments of cardiac structure and function [282]. These AI tools can recognize subtle patterns that may indicate early stages of HF, improving diagnostic accuracy and enabling intervention before the disease’s deterioration [282]. Additionally, AI-driven ECG analysis can detect HF earlier by identifying electrical changes in the heart that might not yet be visible through imaging [282]. As a treatment strategy, AI plays a key role in monitoring HF patients, both in-hospital and remotely. AI models integrated with in-hospital monitoring systems can predict clinical deterioration by analyzing patient vital signs, detecting early signs of HF decompensation, and alerting clinicians to intervene before the condition worsens [282]. Moreover, AI-enabled wearables and implantable devices allow for continuous, real-time monitoring of heart function, such as detecting fluid buildup or changes in heart rhythm [282]. These tools help to predict heart failure exacerbations and guide personalized treatment plans, leading to timely medication adjustments or interventions that prevent hospital readmissions and improve long-term outcomes [282]. AI’s predictive capabilities in both hospital and outpatient settings are increasingly crucial for managing HF effectively.

## 8. Limitations

Although metabolic modulation has emerged as a potential therapeutic approach in HF, with drugs like perhexiline, trimetazidine, and ranolazine showing initial promise, caution is warranted when interpreting the results from these clinical trials. The evidence from studies using these agents in HF remains inconclusive, and thus it is difficult to classify them as truly novel therapeutic strategies. For instance, the concerns raised by Nassiri et al. in their recent meta-analysis [193] emphasize the significant limitations of existing trimetazidine trials, such as small sample sizes, lack of long-term data, and methodological inconsistencies, which undermine confidence in the results. Furthermore, while ranolazine has shown efficacy in chronic coronary ischemia, it is important to recognize that this condition differs from heart failure in its pathophysiology. As such, conclusions drawn from studies on ranolazine in ischemic conditions cannot be directly applied to heart failure management. This distinction is critical, as conflating the two conditions risks overgeneralizing the potential benefits of metabolic modulators in HF without robust evidence to support their efficacy in this specific clinical setting. Many of the discussed metabolic modulators such as trimetazidine, perhexiline, ranolazine, and SGLT2 inhibitors are already in use, aiming to improve cardiac energy metabolism by targeting mitochondrial function or substrate utilization. Their mechanisms are designed to optimize energy production or utilization within cardiac cells. However, they are often used alongside standard HF treatments rather than replacing them and they offer complementary benefits. Neurohormonal modulation remains the cornerstone of HF management, as it directly addresses the systemic mechanisms of heart failure progression. These metabolic therapies provide a valuable complement, particularly for patients who may benefit from enhanced cellular energy production or have persistent symptoms despite optimal standard care.

Another significant concern is how we could move from theory to the implementation of newer imaging techniques and artificial intelligence in clinical practice. While promising, newer imaging techniques and artificial intelligence are in the early stages of clinical application, with ongoing research focused on enhancing their accuracy, accessibility, and practicality. Currently, techniques like PET and magnetic resonance spectroscopy have shown potential in assessing myocardial energetics, yet limitations such as cost, complexity, and the need for specialized equipment have confined their use to research settings. To bridge this theory-to-practice gap, further studies are essential to validate these methods across broader patient populations and develop standardized protocols. Integrating artificial intelligence could help to streamline data interpretation, making these technologies more user-friendly and clinically relevant. Collaborative efforts across cardiology, biomedical engineering, and computational sciences are likely the next steps in translating these advanced diagnostics into practical tools, moving us closer to individualized heart failure management based on energetic status.

## 9. Conclusions

The heart failure starvation model remains highly relevant, warranting greater attention as a means to better understand and diagnose the pathophysiology of heart failure syndrome based on underlying mechanisms rather than phenotypic presentation. Embracing this model may also help to expand our therapeutic approaches.
